# Global distribution and gap analysis of equine housing research: The findings so far and where to go next

**DOI:** 10.1017/awf.2024.64

**Published:** 2024-12-11

**Authors:** Theresa Robertson, Ella Thomas, Gareth Starbuck, Kelly Yarnell

**Affiliations:** School of Animal & Environmental Science, Nottingham Trent University, Southwell NG25 0QF, UK

**Keywords:** Animal welfare, global, horse, husbandry, knowledge exchange, stable

## Abstract

As a free-ranging, social species, the housing of horses (*Equus caballus*) may limit their opportunity to display natural behaviour, compromising well-being. This review records and presents studies that have investigated horse housing design, evaluates the location and number of studies carried out to date, and reports the methods used to assess impact on equine well-being. A Boolean search was conducted in two databases: Web of Science and Scopus, filtered according to Preferred Reporting Items for Systematic reviews and Meta-Analyses (PRISMA) protocol, resulting in 60 peer-reviewed papers for evaluation. Key findings are that a significant amount of work to date has been carried out in Europe and the USA, and the frequency of horse housing studies has steadily increased over the last 33 years, with 52% of them occurring in the last eight years. Health and welfare measures indicate benefits of housing horses in more natural management systems, particularly with conspecifics. Generally, the studies reviewed were only conducted in the short term, therefore future research should aim to increase the length of time over which housing is evaluated, particularly to ensure studies continue beyond an adaptation period. The review also highlights a requirement for more standardised methodology in housing welfare evaluation to allow for more meaningful comparisons to be made. Studies seeking to improve horse welfare in existing housing systems, in the face of limited space or other management constraints, are of high value to the end user and are encouraged. The studies reviewed here represent a significant and diverse body of work from which gaps in knowledge and future research directions can be determined.

## Introduction

Horses (*Equus caballus*) are social animals that evolved to spend most of their time with conspecifics (Christensen *et al.* 2002), roaming vast areas of open land (Green & Green 1977). Housing horses may limit their opportunity to display this natural behaviour, compromising well-being (Yarnell *et al*. 2015). Due to this, the way horses are housed has received considerable research attention, particularly over the last twenty years.

As a free-ranging, social species that can cover between 3–30 km daily under natural conditions (Goodwin 2002), time at pasture is important for horses to allow free movement and contact with conspecifics however, during winter months surfaces may become damaged from the activity of horses and require periods of rest (Furtado *et al*. 2022). By having alternate accommodation for horses, it allows time for ground to recover and grass to grow. Stabling may also be required during conservative management of injury, limiting a horse’s movement and aiding recovery (Peeters *et al.* 2024).

With the use of stables comes the choice of design. Traditionally, in the UK, horses are single housed with three full walls and a front wall with a half door, allowing the horse visual access. This provides a typical internal floorspace of between 3 to 4 m^2^, often guided by the size of the intended occupant. The layout of stabling is often horseshoe-shaped with stables looking into a courtyard, allowing visual access to horses housed around them. Other designs include American barn, where rows of stables are inside a barn providing further protection and rows of stables opposite each other, allowing horses visual contact with conspecifics. The standard size of individual stabling is a fraction of the space which the horse has evolved to live in, resulting in a decrease in ranging movement (Maisonpierre *et al*. 2019). Stabling limits the ability of horses to engage in natural behaviours such as grazing and conspecific interaction. When these behaviours are suppressed, horses may experience a decrease in well-being and, as such, look for ways to cope, including the display of stereotypic behaviours (STB) (Nicol 2010). Historically, it has been reported that individuals who display such behaviours are not coping with their environment as well as those who do not display the behaviours. However, research now suggests that those who display STB are seen as “*pro-active copers*” and those who do not display STB are “*passive copers*” (Budzyńska 2014). As such, a lack of such behaviour does not necessarily suggest adequate welfare and positive markers should receive equal attention (Lesimple 2020). In addition to behavioural findings, physiological measures have been used when assessing welfare including glucocorticoids and their metabolites (Möstl & Palme 2002), heart rate and heart-rate variability (Stucke *et al*. 2015). Housing has been adapted in some cases to attempt to accommodate the species-specific needs of the horse, whilst still maintaining the advantages of stabling. Enrichment devices have been developed to encourage trickle feeding and reduce boredom, including feeding balls (Henderson & Waran 2001), and items hung from the ceiling (Bulens *et al*. 2013). Walls have had grilled windows inserted between adjacent stables, allowing for visual, some tactile and olfactory communication with neighbours (Cooper *et al.* 2000). Horses have shown that they are willing to work for company (Lee *et al*. 2011), which has resulted in increased interest in equine social housing. Despite the significant attention that has been paid to this subject area, findings are mixed, potentially from a diversity of housing and the variety of behavioural and physiological methodologies used.

This paper aims to systematically review the current literature on equine housing design and summarise the findings reported regarding the impact on equine behaviour and welfare. We also summarise the geographical location, methodologies used and the design of horse housing that has been tested to date. We hope this will offer a base of collated information to identify knowledge gaps, suggest improvements in research design and facilitate inter-study comparisons.

### Systematic identification of papers to include

A literature search was conducted during March 2023 using two databases: Web of Science (https://www.webofscience.com/wos/woscc/advanced-search) and Scopus (https://www.scopus.com/home.uri). Additionally, a snowball search was conducted to capture any missing relevant literature by screening the reference lists of identified papers for any further publications not identified via the initial searches.

The Boolean search terms were chosen to encourage the return of papers relating to the effects of housing on equine behaviour and welfare. The search terms were selected in accordance with population, intervention, comparison and outcome (PICO) structure, which is a specialised framework used to facilitate literature review.

For Web of Science (WoS), the following BOOLEAN search was conducted:

(hous* OR stabl* OR stall OR box OR “social* hous*” OR “group hous*) AND (equi* OR horse* OR pon* OR “equus caballus”) AND (behav* OR response* OR activity OR rest* OR “rest behavio?r” OR recumben* OR “sternal* recumben*” OR “lateral* recumben*”) AND (welfare OR wellbeing OR stress OR “stress-behavio?r”)

Scopus required extensions of words, resulting in the following search string to be used:

(housing OR house OR stable OR stabling OR stall OR box OR “social housing” OR “socially housed” OR “group housed” OR “group housing”) AND (equine OR equid OR horses OR horse OR pony OR ponies OR “equus caballus”) AND (behaviour OR response OR responses OR activity OR resting OR “rest behavio?r” OR recumbency OR recumbent OR “sternal recumbency” OR “sternally recumbent” “lateral recumbency” OR “laterally recumbent”) AND (welfare OR wellbeing OR stress OR “stress-behavio?r”)

### Extraction of data from selected papers

**Figure 1.**
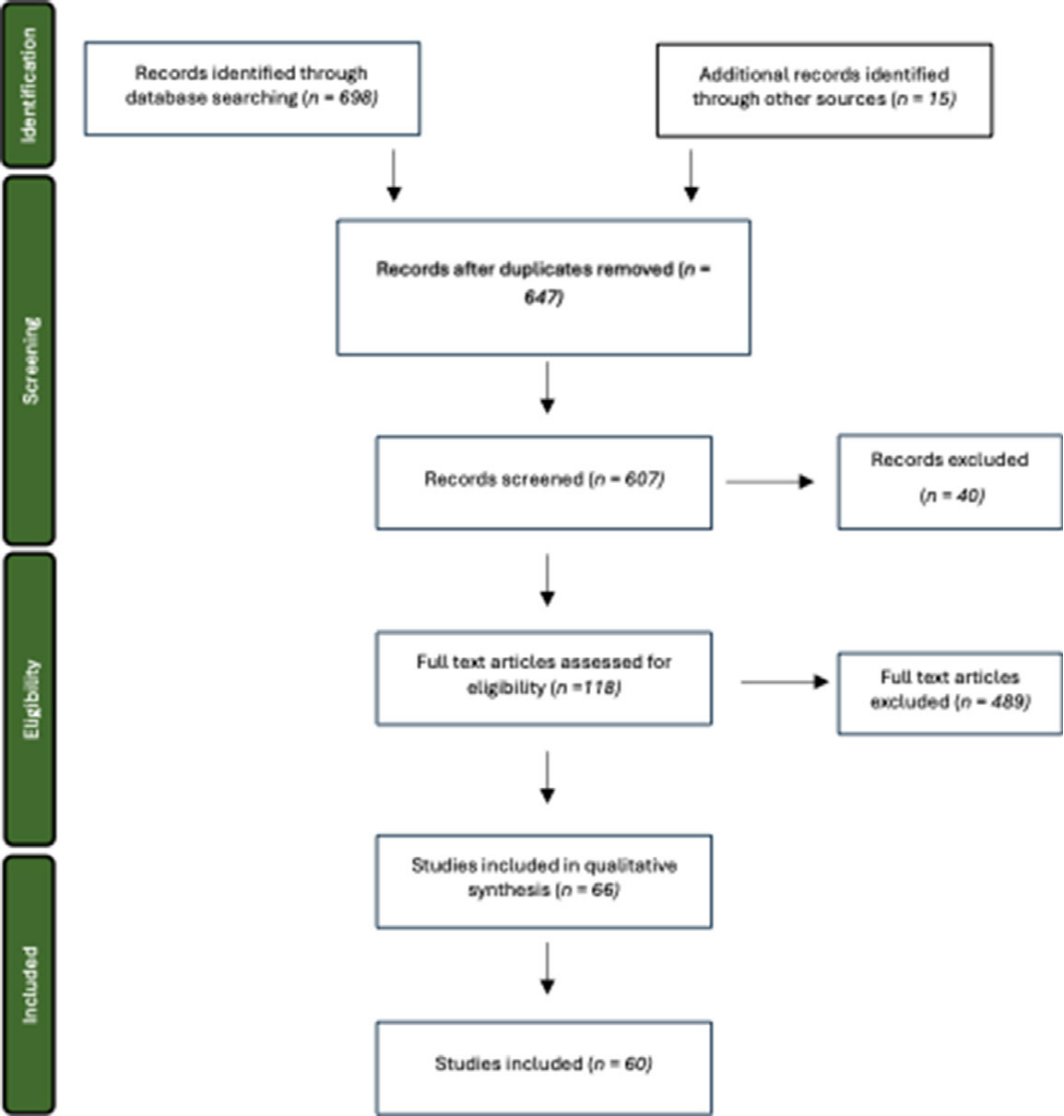
Stages of the PRISMA protocol used to process all horse housing-related publications identified via the database search. The number of publications included and excluded at each stage are shown resulting in sixty publications remaining for a full evaluation and extraction of data.

Inclusion and exclusion criteria (Table 1) were created to aid in identifying relevant papers. In conjunction with this, preferred reporting items for systematic reviews and meta-analysis (PRISMA) protocols were utilised as the standard format for systematic reviews (Figure 1).

**Table 1.** Inclusion and exclusion criteria applied to horse housing publications to determine if they were taken forward for a full evaluation and extraction of key data

Inclusion	Exclusion
Assessment of horse housing systems	Enrichment devices (e.g. mirrors, feed balls etc)
Horse *(Equus caballus)* is focal species	Studies focused on donkeys, mules, zebras or other equids
Studies utilising behaviour, physiological measures, or factors that could impact health of horses	Studies that assessed housing designs but examined impact on any other variables, e.g. sedation effects.
Peer reviewed	Dissertations, abstracts, conference presentations, books

Reports assessing housing design for horses were included for a full screening, including those that assessed the impact of housing on equine behaviour, physiology, or other factors related to equine well-being. Publications that did not assess housing management or design were not included. Papers were read in full, and the following information was retained: 1) Type and size of housing assessed; 2) Impact of housing on behavioural, physiological and other parameters; 3) Duration of study; 4) Location the research was conducted; and 5) Year the study was published.

### Analysis of publications

Each paper was assigned to one of five generalised experimental design categories: 1) Indoor versus outdoor housing; 2) Indoor housing, equal group size; 3) Indoor housing, unequal group size; 4) Natural housing; and 5) Survey and other designs (Table 2). For each paper, data were extracted and recorded, and findings summarised, including the methods used and the key findings on equine behaviour, physiology and any other additional measures. It was also noted for each paper whether methods were used in combination or in isolation.

**Table 2.** Definitions for the five generalised experimental design categories that were used to group publications including indoor vs outdoor housing, indoor housing with an equal group size of horses, indoor housing with an unequal group size of horses, natural housing and studies that utilised a survey

Term	Definition
Indoor housing vs outdoor housing	Indoor housing compared to an outdoor environment, including field, pasture, paddock. Treatments can range from one horse to multiple and include turn-out or exercise vs no turn-out or exercise.
Indoor housing equal group size	Stables comparing two or more indoor architectures with matched group size, e.g. all individual or all group housing.
Indoor housing unequal group size	Individual indoor housing compared to stables containing two or more horses.
Natural housing	Publications investigating the effects of novel stable designs as stand-alone systems, that aim to provide a more natural way of housing horses.
Survey and other designs	Publications where surveys were distributed to working yards or where research was conducted on a working yard and therefore there was limited control or mixed stable design.

### Geographical assessment

The location of every included study was extracted from the method of each paper, summed by country and plotted using ArcGIS v10.8 software, to visualise the spread of equine housing research globally that is included under the terms of this review, as well as identify regions of the world where housing research under the terms of this review is limited or absent.

## Results

### Description of literature

A total of 698 papers were identified that focused on the effect of housing design on equine welfare from Web of Science and Scopus. In addition, 15 papers were manually added from the snowball search. Six reviews came back in the searches, any publications that fitted the criteria that did not appear from the Boolean search were added and reviews were removed to prevent duplication of data. Upon PRISMA protocol application, 60 papers remained.

### Geographical results

**Figure 2.**
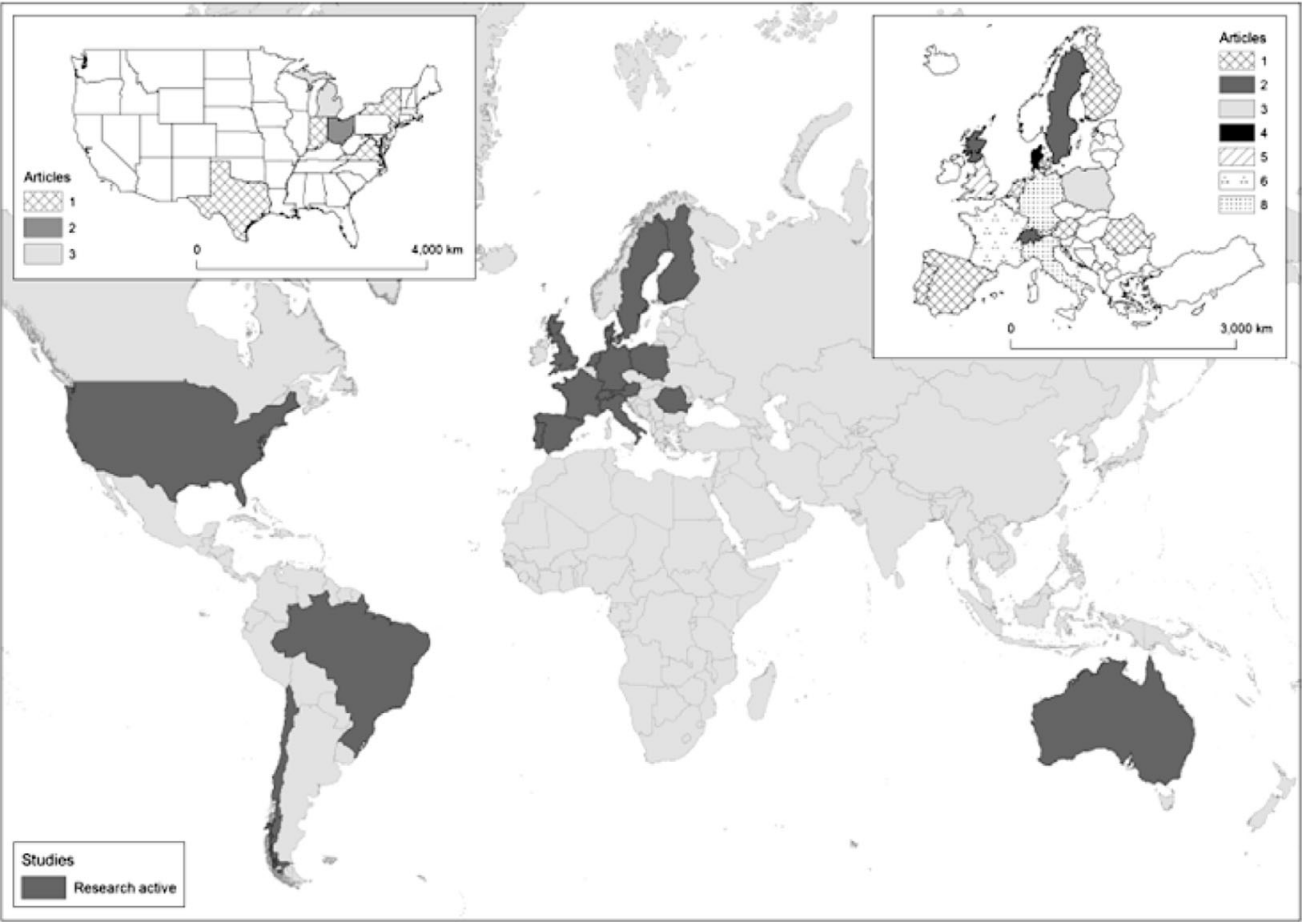
Global distribution of studies into horse housing (main map) highlighting work carried out in North and South America, Europe and Australia. Figure also details the distribution and number of studies across North America (top left) and Europe (top right). Location data were taken from the method section of each paper regarding where the study was carried out.

From the studies included in this review, 92% were carried out in Europe (n = 46) and the USA (n = 9) with the remaining studies carried out in South America (n = 3) and Australia (n = 2). Figure 2 provides a visual representation of the location of the equine housing studies.

### Year of publication

**Figure 3.**
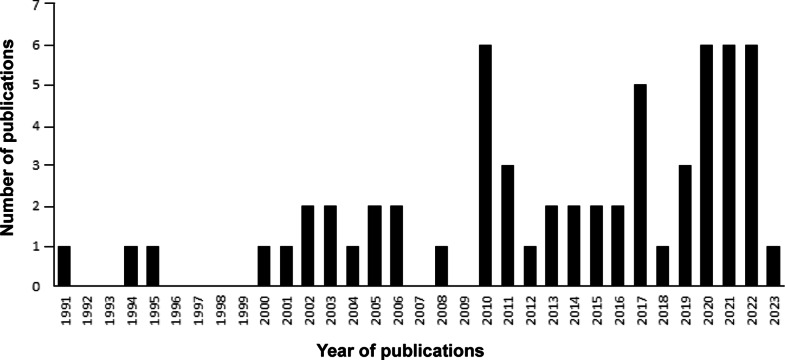
Number of housing publications included in the review that have been published over the last thirty-three years (1991–2023).

Publications included in the review span 33 years from 1991 through to early 2023 (Figure 3). Both physiological measurement and behavioural observations are included across the entire time range of studies. Air quality has been included as a measure in three studies in 2010 (Berndt *et al*. 2010; Walinder *et al*. 2011; Whittaker *et al*. 2010), one study in 2011 (Millerick-May *et al.* 2011) and one in 2012 (Millerick-May *et al.* 2012).

### Indoor vs outdoor housing

Thirty-two publications compared an individual indoor housing system to an outside system which was either an increased exercise regime, additional turn-out, permanent housing in a pasture or management on a reserve. (Table 3).

**Table 3.** Summary of studies that investigated indoor versus outdoor housing with information on lead author, year of publication, housing designs included in the research, the size of the housing, the length of time over which the study was conducted and the results according to variable measured which were behavioural, physiological or health related parameters. Where paddocks have been reported as hectares, this has been converted to metres squared (m2) to allow for easier comparison*not all studies are included, some are discussed rather than summarised here.

Author	Housing design	Stable / Housing size	Length of study related to housing	Behaviour	Physiology or impact on health
Mal *et al*. (1991)	Isolation stall Social stall Pasture group	3.6 m × 3.6 m 4.2 m × 6.0 m 45,000 m^2^	Unclear	_	Increased blood haemoglobin and depressed MCHC in isolated treatment
Houpt *et al*. (2001)	Individual tie stall 30 min exercise per day Individual tie stall 30 min exercise every 14 days	1.2 m × 3.6 m	6 months	Confined horses: Rebound locomotory behaviour	Confined horses increased cortisol. *9/16 did not lie down for entire study (6 months), 13/16 collapsed at least once
Christensen *et al*. (2002)	Single housed Groups of three horses in each stable Paddock *Stallions	3.6 m × 2.5 m 5.6 m × 4.8 m 20,000 m^2^	9 months	Single housed: Increase in aggressive behaviour. Group housed: Increased agonistic interaction. Group housed likely to have a former group mate as their nearest neighbour in paddock	_
Heleski *et al*. (2002)	Single housed Groups of 3 in paddock	3.6 m × 3.6 m 992 m^2^	56 days	Single housed: Increased aberrant behaviour Paddock housed: increased natural behaviour.	Faecal cortisol no treatment effect
Harewood & McGowan (2005)	Single housed Six horses in paddock	5.0 m × 5.0 m 30 m × 35 m	24 h per treatment	Increased stress related behaviour in single housing	HR & salivary cortisol no treatment effect
Nicol *et al*. (2005) *Weaning method predominantly investigated diet	Barn weaned Paddock weaned *Not clear if horses remained in these treatments until behavioural tests were carried out	Not stated	Initial tests within 3 h of removal of mare, 3 × 15-min observations Additional tests 2 months post-weaning	Barn weaned more frequent defaecation, walking and investigating. More frequent and longer pawing and less time foraging than paddock weaned	_
Chaplin & Gretgrix (2010)	Full stabled (no turnout) Part stabled (turn out at night) Yard (pairs) Paddock (pairs)	3.5 m × 3.5m 3.5 m × 3.5 m 4,000 m^2^ 10,000 m^2^	7 days in each treatment (6 days acclimatisation, 24 h recording)	Significantly more active behaviour in yard and paddock No treatment effect on recumbency behaviour Rebound behaviour post-confinement	_
McGorum *et al*. (2010)	Single housed conventional Single housed low dust Pasture	3.4 m × 2.6 m (no vents) 4.5 m × 4.3 m (vents & dust free management) *Only one horse used.	4–10 h per treatment	_	Increased airborne endotoxins (measured via breathing zone assessment of horse) in conventional box.
Whittaker *et al*. (2010)	Single housed with four bedding / forage systems Paddock	Not stated	3 weeks per treatment	_	Increase in ambient ammonia concentration in individual housing. (condensate of equine exhaled breath)
Lesimple *et al*. (2011)	Single housed Paddock	Not stated	Usual Housing	Positive behavioural responses in paddock housed	_
Erber *et al.* (2013)	Single housed Paddock	3.0 m × 3.7 m	4 days before to 5 days post transfer to box	Increased locomotion in paddock housed	Individual housed increase in salivary cortisol and HR
Yarnell *et al*. (2015)	Individual no contact Individual contact Paired housed indoor Group housed outdoor	3.0 m × 3.6 m 3.0 m × 3.6 m 10.9 m^2^ 60 m × 20 m	Five days in each treatment	Increased STB in single housed Improved handleability in group housed	Increased faecal glucocorticoids single housed
Giannetto *et al*. (2016)	Single housed Single Paddock	4.0 m × 4.0 m 1,500 m^2^	*Usual Housing	Stress related locomotion in TB in single housed	_
Pessoa *et al*. (2016)	Single housed Single housed with individual turnout	3.0 m × 3.0 m 53 m^2^	16 days	Increase in calm behaviours, social interactions, and vocalisation in turnout group	Increased HR, and cortisol in individual housed group
Henry *et al*. (2017)	Single housing Paddock housed	3.0 m × 3.0 m 10,000–20,000 m^2^	3 years	STB, aggression increased and latency to approach decreased in single housed horses	_
Junkkari *et al*. (2017)	Single housed Group housed with shelter	Not stated 3–19 m^2^ per foal	2 examinations 58 days apart	_	Smaller space increased chance of diagnosis of clinical respiratory disease
Krakowski *et al*. (2017)	Single housed Free ranging in reserve	Not stated	Usual Housing	_	Improved cell defence markers in free ranging horses
Schmidt *et al*. (2017)	Single housed Paddock	3.0 m × 6.0 m Not stated	25 weeks	_	Shorter guard hair / slower regrowth in indoor group. No treatment effect in plasma testosterone or cortisol No treatment effect on fertility parameters between groups.
Giannetto *et al*. (2018)	Single housed Paddock	3.0 m × 4.0 m 10,000 m^2^	Locomotion recorded for 7 days	No treatment effect on locomotory behaviour	_
Connysson *et al*. (2019)	Single housed Paved paddock	3.0 m × 3.0 m 3,200 m^2^	21 days per treatment	_	Improved appetite, recovery in paddock. No treatment effect on heart rate, plasma lactate, plasma urea, or total plasma protein concentration
Falomo *et al*. (2020) *authors report several potential confounding factors	Single housed post weaning (mares) Paddock post weaning (mares)	Not stated	7 days pre weaning to 30 days post weaning	Increased vocalisation in single housed	No treatment effect in milk, salivary and hair cortisol
Marr *et al*. (2020)	Single housed Pasture	3.2 m × 3.5 m 59,000 m^2^	Between 1 week and two months	Laterality shift to left in single housed (within one week)	Increased faecal glucocorticoid in single housed No treatment effect on faecal IgA
Molinari *et al*. (2020)	Single housed no contact Single housed outside / con-specific access Paddock group (2–5) Pasture group (min7)	Not stated	Usual housing for at least six months	STB highest in single housed no contact	No treatment effect on blood parameters for oxidative stress
Ruet *et al*. (2020)	Single housed Pasture	3.0 m × 3.0 m Not stated	Single usual housing 3 days pre pasture to 3 months post	No STB, or aggressive behaviour toward humans in pasture housed Increased STB in single housed	_
Arena *et al*. (2021)	9 barns with varying ranges of restriction in single housing and outdoor paddocks	8 m^2^–24 m^2^ stalls 60–1,500 m^2^ Paddocks	Usual housing *samples taken over 1 month	Increased behavioural pathologies as restriction increased	No treatment effect in hair and plasma cortisol.
Jastrzębska *et al*. (2021)	Single housed Free ranging	3.0 m × 3.0 m	*Usual housing	Less impact of isolation on group housed lncreased latency to approach humans in free ranging horses	
Mach *et al*. (2021)	Single housed Pasture	3.0 m × 3.0 m Not stated	1.5 months at pasture single housed time?	STB increased in single housed	Improved faecal microbial count in pasture group
Popescu *et al*. (2022)	Tie stalls Padddock	Not stated 150,000 m^2^	Three months from release to pasture to study end	Improved handleability when horses at paddock	Normalised BW, lower rest related lesions, reduced joint swelling when in paddock
Schmucker *et al*. (2022)	Pasture Social group change pasture Single housing	22 000 m^2^ 3.2 m × 3.5 m	Six weeks Eight days Unclear but at least two weeks	Increased aggressive behaviour in single housed	Reduced immunological markers in single housed Increase in cortisol in single housed *No difference in change of group composition treatment

* May be study duration and not necessarily time spent in housing

### Behavioural assessment methods

Ten studies measured only behaviour. Giannetto *et al*. (2018) reported a breed-specific locomotion response to housing design, with no effect on locomotion for Standardbreds and Italian saddle horses, but an increase in locomotion parameters for Thoroughbreds associated with individual box housing compared with paddock housing. The authors attribute this to differences in breed temperament with the character of Thoroughbreds contributing to the increased locomotor activity when housed in confined conditions. All other studies that assessed behaviour alone, reported a positive impact of outdoor housing on equine behaviour. No studies reported a positive impact of single housing on equine behaviour and there were no mixed findings.

### Physiological assessment methods

Seven studies measured only physiological parameters. Of these, six reported positive effects of outdoor housing, including one study reporting positive effects of outdoor housing on post-exercise recovery (Connyson *et al*. 2019). One study reported no treatment effects of housing on physiology and no studies reported positive effects of single housing on equine physiology.

### Physiological and behavioural assessment methods

Thirteen studies measured both behaviour and physiology. Of these, eight reported increased stress-related behaviours in single-housed horses that are supported by the physiological parameters studied and five studies reported stress-related behaviour in single-housed horses but no effect of housing in the physiological measures recorded. No studies report confounding behavioural and physiological findings where one parameter has positive and one has negative changes. In addition, one study investigated air quality alone and reported that mean endotoxin concentration measured in the breathing zone of stabled horses is more than eight-fold higher than that of horses kept on pasture (Berndt *et al*. 2010). Stomp *et al*. (2018) aimed to validate snorting as a measure of positive welfare and reported increased snorting in paddock housing when compared to single housed. No studies reported positive effects of single housing.

Stables with grills in the walls between adjacent stables were the highest level of confinement in five of the 23 publications (Christensen *et al*. 2002; Erber *et al*. 2013; Marr *et al*. 2020; Ruet *et al*. 2020; Schmucker *et al*. 2022). Other studies in this category included wooden panels that allowed visual and physical contact (Erber *et al*. 2013) and pipe rails which allowed for olfactory, visual and auditory communication (Houpt *et al*. 2001). A slightly higher level of confinement was seen in Mach *et al*. (2021) where visual contact was possible, but no physical contact. Stall doors that opened into a barn corridor were used in Junkkari *et al*. (2017). Solid partitions were used for three studies (Heleski *et al*. 2002; Berndt *et al.* 2010; Arena *et al*. 2021). Two designs aimed to assess environmental effect by having windows for ventilation (Giannetto *et al*. 2018) and windows for daylight (Schmidt *et al.* 2017). One study put horses in isolation with no contact with conspecifics (Mal *et al*. 1991).

### Indoor housing, equal group size

A total of seven studies compared housing designs between indoor stable designs where the number of horses accommodated in each design was the same or carried out their whole study indoors in a single stable. Four studies measured behaviour alone, all these studies report positive behavioural responses to larger stables, social boxes, or increased conspecific contact. The remaining studies measured physiological parameters. Table 4 provides further details of these studies.

**Table 4.** Summary of studies that investigated indoor housing with equal group sizes of horses with information on lead author, year of publication, housing designs included in the research, the size of the housing, the length of time over which the study was conducted and the results according to variable measured which were behavioural, physiological, health-related parameters or additional measures

Author	Housing design	Stable / Housing size	Length of study	Behaviour	Physiology	Additional measures
Cooper *et al*. (2000)	Single housed front half door open Single housed front and back half door open Single housed back half door open Singles housed front and one side grill open Single housed front, rear and both side grills open	3.6 m × 3.6 m	Five days in each treatment	Decrease in weaving in the front and side and all four open treatments All treatments less head nodding compared to front only treatment		
Raabymagle & Ladewig (2006)	Large Box Small Box	2.5 × height of the horse^2^ m^2^ 1.5 × height of the horse^2^ m^2^	5 days adaptation the 3 days testing	Significant increase in sternal recumbency in large box	_	_
Millerick-May *et al*. (2011) *Also explored location and orientation of barns	Individual stalls with roll up sides, open front, facing outdoors & high ceiling Individual stalls with closed front and high closed window As 2 but with windows open	34 stalls 128 stalls 128 stalls	Measurements taken once per day for three days per treatment	_	_	Treatment 2 had significantly higher levels of airborne particles
Wålinder *et al*. (2011)	Mechanical ventilation intervention	12 m × 30 m indoor barn with 24 individual stables	Dependent on measure 4–37 h		No treatment effect on human pulmonary function Reduction in equine mucus	Reduction in C0_2_ levels Reduction in ammonia levels
Millerick-May *et al*. (2012)	Individual stalls with roll up sides, open front, facing outdoors & high ceiling Individual stalls with closed front and high closed window As 2 but with windows open	34 stalls 128 stalls 128 stalls	Measurements taken once per day for three days per treatment Endoscopic tracheal examination and wash as above	_	Mucus score highest in treatment 2. And associated with high concentration of large particles. 4× greater incidence of high neutrophil concentration in treatment 2 and 3 associated with small particles.	Treatment 2 had significantly higher levels of airborne particles. Particle concentration higher and more sustained in treatment 2 and 3.
Lesimple *et al*. (2019)	Visual contact only Visual, and tactile contact	3.1 m × 3.1 m 3.0 m × 3.0 m	Usual stable for at least 6 months	Lower excitation behaviours and increased rest behaviour and snorting in treatment 2.	_	_
Grzyb *et al*. (2022) *refer to publication for a description	Runner stables* group housed (deep littered) Box stables (daily cleaning)	283–386 m^2^ between 12–18 horses 395–450 m^2^ between 17–20 horses	Two years			Higher bacterial and particulate contamination in treatment 1 Higher summer temperature in treatment 2.
Gmel *et al*. (2022)	Conventional stable Social stable	3.0 m × 3.0 m limited contact 3.0 m × 3.0 m with partition that allows physical contact	4 weeks conventional, 4 weeks social then 8 weeks conventional	Decrease in unwanted social interactions whilst in social box	_	_

### Indoor housing unequal group size

A total of six studies compared housing designs between indoor stables where the number of horses accommodated in each design was different. Three of these studies investigated behaviour alone. One study reported increased latency to approach a human in single-housed horses and no treatment effect in the group-housed horses during an arena test, as well as other positive behavioural indicators in single-housed horses (Søndergaard & Halekoh 2003).

A second study, investigating the housing of breeding horses, reported increased fear response to a novel object in group-housed horses as well as higher numbers of lesions from horse-to-horse interactions (Sanchez *et al*. 2020). The remaining study reported reduced aggression in group-housed horses (Søndergaard & Ladewig 2004). Two studies utilised both behavioural and physiological measures. One reported negative consequences of weaning foals in pairs for both behavioural and physiological indicators (Hoffman *et al*. 1995) and the second study reported both behavioural and physiological indicators of stress in single-housed horses (Visser *et al*. 2008). The final study utilised physiology alone and reported no treatment effect. Table 5 provides details of these studies.

**Table 5.** Summary of studies that investigated indoor housing with unequal group size of horses with information on lead author, year of publication, housing designs included in the research, the size of the housing, the length of time over which the study was conducted and the results according to variable measured which were behavioural and physiological parameters

Author	Housing design	Stable / Housing size	Length of study	Behaviour	Physiology
Hoffman *et al*. (1995) *weaning in foals	Single stalls Paired stalls	Unable to access full article.		Increased signs of stress in paired housing	ACTH challenge lower (adrenal depletion) in paired housed
Søndergaard & Halekoh. (2003)	Single Housing Groups of three	3.0 m × 3.0 m 5.0 m × 5.0 m	2 years	Increased latency to approach human in single housed (group housed no effect) Single housed less restless, more explorative and less vocalisations. *in arena test	_
Søndergaard & Ladewig (2004)	Single housed Groups of three	3.0 m × 3.0 m 5.0 m × 5.0 m	6 months old until 2 years old.	Group housed ‘passed’ more behavioural tests Single housed bit their trainer more	_
Visser *et al*. (2008)	Single housed Paired housing	3.2 m × 3.2 m 48 m^2^ per pair	12 weeks	Stress related behaviour more frequent in single housing	Stress induced elevations and via ACTH challenge.
Aurich *et al*. (2015)	Single housed (access to paddock or ridden) Group housed (access to a paddock)	Not stated	6 months	_	No treatment effect on salivary cortisol
Sanchez *et al*. (2020)	Single housed stallions Group housed broodmares (pasture access)	2.81 m × 2.86 m 15,000 m^2^ (43 mares)	Usual housing	Group housed had increased fear response and lesions *Group housed had lower BCS	_
Kjellberg *et al.* (2022)	Single boxes at night & paddock during day Group housed (n = 10) with access to shelter As 2 but access to two shelters As 2 but with access to larger lying area	3.2 m × 3.2 m 80 m^2^ (shelter lying area of 8 m^2^) 80 m^2^ (shelter lying area 18 m^2^) 80 m^2^ with lying area of 28 m^2^	7 days acclimatisation then 3 days data recording	Horse spend more time with more bouts of lateral recumbency with increased lying space.	_

Two publications investigated single housing compared to paired housing (Hoffman *et al*. 1995; Visser *et al*. 2008). Four studies compared single and group housing (Søndergaard & Halekoh 2003; Søndergaard & Ladewig 2004; Aurich *et al*. 2015; Sanchez et al. 2020).

Descriptions of housing design were varied. One study had three solid walls with the front wall being a half grid allowing visual and auditory communications (Visser *et al*. 2008). Vertical bars were installed to create a social box in another study, allowing for visual, audible, olfactory and tactile communication, but no physical interaction (Søndergaard & Halekoh 2003). Similarly, Søndergaard and Ladewig (2004) reported visual, audible, olfactory and tactile communication in their stable design.

### Natural housing

Six publications investigated the effects of housing designed towards meeting the natural needs of the horse, including increased opportunity for movement and social interactions, whilst maintaining the convenience of traditional management. Table 6 provides a summary of these studies.

**Table 6.** Summary of studies that investigated natural housing with information on lead author, year of publication, housing designs included in the research, the size of the housing, the length of time over which the study was conducted and the results according to variable measured which were behavioural and physiological parameters

Author	Housing design	Housing size	Length of study	Behaviour	Physiology
Rose-Meierhöfer *et al*. (2010)	Three open barns Two active barns Paddock	600–1,000 m^2^ 2,000–4,500 m^2^ 800 m^2^	4 weeks	Higher activity level in Active barn 2	_
Placci *et al*. (2019)	Conventional management Natural management	Single housing with no contact, prescribed feeding, shod and clipped. Ridden in bit Paddocks (min 12 h per day), free movement, adlib fed, free thermoregulation, barefoot, no bit.	3 months	_	Hair dehydroepiandrosterone higher and cortisol lower in natural management horses
Marliani *et al*. (2020)	‘Ethological stable’ or Big Box©	1,000 m^2^ system incorporating variety of surfaces & tracked system.	7 months (9,920 min)	Time budgets closely reflect those reported in feral horses	_
Mazzola *et al*. (2021)	Single housed (turnout available through day) Paddock Housed Natural management in land, wood and olive groves, natural water source	4.0 m × 4.0 m 40,000 m^2^ wild area, size not stated	8 months	_	Paddock group higher hair cortisol
Hildebrandt *et al*. (2021)	‘HIT’ Active system	10 000 m^2^	227 days	Assessed movement of horses around footprint and advised spacing of resources	_
Marliani *et al*. (2022)	Single housing Natural boarding system ‘Ethological stable’ as in study above	3.0 m × 3.5 m 60 000 m^2^ with natural features and range of surfaces		‘Ethological stabled’ horses registered a more ‘optimistic’ affective state	Inconclusive findings of faecal and hair cortisol

### Survey based and other housing studies

Publications included in this section were those that either utilised a survey distributed to horse owners investigating relationships between housing and equine traits, or studies that conducted research in working yards and therefore stables lacked common characteristics.

Rosenthal *et al*. (2006) compared respirable airborne particulate levels in a range of horse barns and found them to vary greatly depending on season. Hotchkiss *et al*. (2007) provided extensive demographic information but no behavioural observations or physiological measures, we have included findings here as they show that 58% of horses surveyed were turned out 24 h per day in the UK, 72% were part stabled with the majority turning out 24 h in summer. The remaining studies all utilised behavioural observations with no physiological assessment which is to be expected for largely survey-based studies.

Hockenhull and Creighton (2014) undertook a survey of horse owners and reported that longer stabling periods and no turn-out resulted in increased risk of handling problems and increased aggression towards humans as well as increased abnormal oral behaviours. They also stated that frustration behaviour increased with visual contact and reduced social contact. Leme *et al*. (2014) and Bachmann *et al.* (2003) also found that there was a greater frequency of abnormal behaviour in horses that spent longer periods of time individually housed, reported by owners via a survey. Waters *et al*. (2010) reported that box-weaned horses had significantly greater risk of developing behavioural problems compared to paddock weaned, when behaviour was observed post-weaning and followed up via a post-weaning survey. Tadich *et al*. (2013) carried out an observational and questionnaire-based study in Chile and reported that 11% of 743 racehorses presented with abnormal behaviour which was lower than most similar studies. The authors suggested that this may be due to stable design in Chile offering increased contact compared to other countries. Finally, Schmitz *et al*. (2020) used a citizen science method to report that time spent walking at pasture was greater in individually housed horses.

## Discussion

### Incidence and global distribution of equine housing research

Horse housing studies have steadily increased in number over the last thirty years, with 56% of studies in this review having been carried out in the last decade. As the studies represent a significant and diverse body of work, it is considered timely to review what has been reported to date, identify gaps in knowledge and suggest future research directions.

The global distribution of studies identified in this review were conducted in western nations and largely show alignment with countries that use horses for leisure and competition purposes with some application in agricultural ranch work. A global record of horse numbers by country is held by the Food and Agriculture Organisation of the United Nations (2024). Whilst data are not submitted by all countries, and a significant amount is based on estimate, the latest extensive records for 2022 show the top six most populous countries, accounting for 56% of recorded horse numbers, are the USA (10.3M; 16.6%), Mexico (6.4M; 10.3%) and Brazil (5.8M; 9.4%), Mongolia (4.8M; 7.7%), Kazakhstan (3.9M; 6.2%) and China (3.7M; 5.9%). There are also records for 36 African countries, representing 11.5% (7.2M) of the total horse numbers. Despite their large populations of horses, many of these countries do not appear to be conducting research into housing design. It may be that horses are stabled less or not at all in these areas however some of these countries do stable horses and account for a large proportion of global horse numbers. Based on this, conducting future studies in horse housing that better represent the global population of horses, with a view to having a greater impact on worldwide horse welfare, should be considered.

### Housing design assessed via behavioural observation and hormonal measures

All but three of the studies in this review reported a positive impact on behaviour, physiology or both parameters, when horses are housed in groups or a more open stable design. The studies that reported a positive impact of single housing all cite improvements in behavioural parameters and could be open to alternative interpretation. It is worth noting that some studies report no change in behaviour or physiology when comparing single- and group-housed horses.

Positive findings from single-housed horses included Søndergaard and Halekoh (2003) who were exploring the effects of housing young horses on horse-human interaction, with a reduced reaction to human interaction interpreted as positive. They reported decreased latency to approach a human in individually housed horses compared with horses housed in groups of three when tested at both 12 and 24 months of age. Single-housed horses approached sooner and were more easily approached by a human than group-housed horses where no effect of handling was observed. Single-housed horses expressed less restless behaviour, more explorative behaviour, and less vocalisation than group-housed horses. Although interpreted as a positive outcome of single housing for the aim of this study, it should also be considered that horses housed as individuals could be seeking interaction, regardless of whether this is conspecific or human, due to limited social contact, whereas the group-housed horses may have a reduced need to seek out this contact due to having companions housed with them. This raises the question of a possible challenge between efforts to increase the welfare standards of equine housing without impacting horse training, particularly with young horses, and warrants further investigation. It is to be hoped that any training benefit derived from individual isolation would be compensated by positive impacts of free movement and conspecific interaction on the temperament, welfare and emotional state of more socially housed horses, as indicated by Lesimple *et al*. (2011).

A second study reported increased fear response to a novel object test for group-housed broodmares in comparison with stabled stallions, however the housing system did not affect the responses to the human approach test (Sanchez *et al*. 2020). The authors suggested that an increased fear response in group-living horses is to be expected as a species-specific behaviour of a herd animal and the ability to express their natural behaviour is a positive indicator of welfare, although the ability to make comparisons between the two different groups of horses here is limited.

The remaining study reported negative consequences of weaning foals in pairs for both behavioural and physiological indicators (Hoffman *et al*. 1995) suggesting that the pairing of foals for weaning is not ideal and alternate methods and housing should be considered in this circumstance.

Positive results were frequently reported in studies that included outside access, regardless of its nature or use. This suggests the importance of natural open space, and the opportunity for horses to express locomotory behaviours. Increase in space was also reported to improve post- exercise recovery in the long term (Connysson *et al*. 2019) and reduce time spent displaying stereotypic and undesirable behaviours, despite no difference in hair or plasma cortisol between study groups (Arena *et al*. 2021). Results have also shown higher cognitive abilities in horses when access to pasture is provided (Lesimple *et al*. 2011). During weaning, separation of mares into individual stables resulted in an increase in negative physical and vocalisation behaviours, when compared to mares placed in a paddock, however both groups of mares had similar elevated cortisol concentrations in response to the weaning event (Falomo *et al*. 2020).

Similarly, in a separate study, weanlings expressed higher vocalisation, decreased eating and aberrant behaviours when housed individually compared with those paddock housed in groups (Heleski *et al*. 2002). There was no significant difference in cortisol metabolites measured between the groups, but levels were elevated four weeks post-weaning in both groups. The four-year prospective study of Waters *et al.* (2010) on 225 weanlings developed these findings further, identifying that weaning foals by confinement was associated with an increased incidence of abnormal stereotypic and redirected behaviours when compared with paddock-weaned foals.

Whilst it appears from the sample of publications in this review that group housing has clear benefits on both behavioural, physiological and environmental parameters, it has also been discussed that the novelty of an altered environment could be responsible for such changes, particularly in studies that show positive behavioural responses (Cooper *et al.* 2000). Longer-term studies would give greater insight into habituation and adaptation when horse housing is changed and the accommodation of an adjustment period in experimental design may allow for reported behavioural and physiological changes resulting more from a change of routine rather than housing design.

Positive indicators of welfare are becoming more popular as a measure of well-being, including recumbency and rest behaviour (Giannetto *et al*. 2018; Stomp *et al*. 2018). Horses are a prey species and are therefore vulnerable when they are recumbent. This suggests that if a domestic horse spends time lying down in the absence of illness or injury, they are comfortable in their environment. However there have been reports of isolated horses making use of time undisturbed by conspecifics to be recumbent in stables (Erber *et al*. 2013), so care is needed when interpreting results of studies investigating rest behaviour.

Lying behaviours in relation to box size have been investigated. Raabymagle and Ladewig (2006) reported that recumbency was higher in a large stable (13.1 m^2^ × 15 m^2^) compared to a small stable (4.7 m^2^ × 6 m^2^) with significantly more time spent in sternal recumbency in the large stable, especially if horses were housed in the small boxes first then switched to the larger housing. This is further supported by research from Burla *et al*. (2017) and Kjellberg *et al*. (2022), which also identified that the larger the lying space given, the longer the horses spent in recumbency. Burla *et al*. (2017) also discovered that recumbency was significantly reduced when no bedding was available, so suitable surface provision for recumbency appears to be another factor that could affect the choice to lie down.

Measurement of locomotory behaviour has been included in several housing studies. Horses are a free-ranging species, therefore ability to roam is important and housing that offers this opportunity may benefit well-being. Walking time and distance increased in horses that spent part of their day housed inside, compared to those housed outside full time (Schmitz *et al*. 2020). It was suggested that those who were kept in stables used their opportunity to move more when outside, therefore creating rebound locomotion. Houpt *et al.* (2001) reported that pregnant mares displayed an increase in cortisol that decreased after two weeks when moved from pasture to stables, but there were no signs of adrenal exhaustion or hypertrophy. Rebound locomotory behaviours were also higher in horses who had limited free movement once turned out, as in Schmitz *et al*. (2020).

Giannetto *et al*. (2016) reported that Thoroughbreds showed significantly more movement in box stables compared to pasture, suggesting an increase in stress, manifesting as box walking. Nature of movement therefore needs to be considered rather than just the movement time or distance in isolation, as not all movement can be regarded as good. In this case, Thoroughbreds were compared with Standardbred and Italian Saddle horses who did not express this increase in locomotion when confined, also highlighting a likely breed and temperament effect that also warrants consideration in experimental design.

Increased cortisol and alert behaviour were reported in horses housed individually for the first time (Harewood & McGowan 2005; Erber *et al*. 2013) and those who had extended periods on full-time pasture before returning to stabling (Ruet *et al*. 2020). This suggests that abrupt changes in management, especially from extensive to confined housing, may be unsettling for horses. It also highlights the importance of maintaining access to turn-out or outside accommodation should housing or shelter design need to be changed, and that some time on pasture can have similar effects to full-time turn-out when it is not available (Popescu *et al*. 2022). Results from surveys supported this, reporting that daily access to pasture and free movement decreased the chance of stereotypic or abnormal behaviours (Bachmann *et al*. 2003; Leme *et al*. 2014).

To compensate for lack of turn-out, paired and group stabling is utilised. Compared to single housing, group housing has reported positive results (Søndergaard & Ladewig 2004; Yarnell *et al.* 2015) however, results for paired accommodation vary. The importance of appropriate pair bonds as well as the situation should not be overlooked as evidence suggests higher aggression can be seen when weaning foals in pairs compared to single housing (Hoffman *et al.* 1995). However, when stabling horses for the first time, Visser *et al*. (2008) reported that stress-related behaviours like neighing, pawing, nibbling and snorting were all displayed more frequently in individually housed horses when compared with paired-housed horses. At the end of the study, 67% of the individually housed horses were seen performing one or more stereotypies. Additionally, cortisol and adrenocorticotropic hormone (ACTH) responses to a corticotrophin releasing factor challenge test were lower for horses in individually housed boxes, suggesting depression in the socially isolated animals caused by a desensitisation of the HPA axis in response to stress-induced elevations in ACTH and cortisol.

No difference in salivary cortisol levels were recorded between group-housed horses in a paddock and horses in individual stabling with access to an individual paddock or daily exercise (Aurich *et al*. 2015). This suggests the importance of outdoor exposure as part of a stabled horse’s routine and that opportunities for movement were enough in this instance in housed horses without constant turn-out as no increase in salivary cortisol was observed when stabled.

It is worth considering that once established, STBs are only reversable within a short time-frame, after which horses may have adequate welfare but be psychologically unable to stop performing the behaviours (Vinken *et al*. 2023). Presence or absence of such behaviours may therefore not be the most appropriate method to assess equine housing unless it is a new method of housing under scrutiny and horses studied do not display signs of STB prior to being introduced to it. Yarnell *et al.* (2015) compared four housing designs which were individual stables with no contact between conspecifics, individual stables with visual and limited tactile contact between conspecifics, paired housing and full turn-out in a group. Results showed presence of STBs in the most restricted housing that was not observed in any of the other housing designs. An increase in faecal cortisol as the level of isolation increased was also reported, and horses were more challenging to handle as social opportunities decreased, directly impacting safety of the horse and human handler.

Lesimple *et al.* (2019) found grilled stables situated inside a barn increased sleeping behaviours compared to stables without a grill, allowing horses to put their head over the door. In addition, all horses demonstrated snort behaviours (classed as a novel method of positive welfare assessment in the study) compared to those in stables with a view of the outside only, where only 42% snorted. There was no difference for time spent conducting STB, but weaving was observed and most prevalent in the more open stables. In comparison, studies have shown that stables inside a barn also reduced the risk of abnormal oral behaviours however, horses being able to see others and not touch them increased frustration behaviour two-fold (Hockenhull & Creighton 2014). Although the results of stereotypical behaviour are contradictory between the two studies, it is worth mentioning that Lesimple *et al.* (2019) had the same observer for all behavioural recordings, but Hockenhull and Creighton (2014) used a survey. In the second part of the Lesimple *et al.* (2019) study, mares were stabled for the first time. Grilled stables resulted in an increase in foraging and resting, standing and recumbency, whereas stables facing outside increased vigilance behaviour.

Cooper *et al.* (2000) reported similar findings, when horses only had access to front and back door openings without a grilled window between stables. There was an increase in standing alert behaviours, but when grills were open between adjacent stables, weaving that had been observed ceased. A similar result was seen when the front door and grills were open, suggesting horses are more interested in interactions with neighbouring horses as opposed to the outside views. Allowing the front door and side grills to be open created similar architecture to that of the open treatment in the study of Lesimple *et al.* (2019) yet different results were recorded. When the front door was open during the study of Cooper *et al.* (2000), a view of a courtyard was visible. However, in the study of Lesimple *et al.* (2019), the stables opened to an arena where the horses worked. Perhaps the difference between the views lead to the difference in findings, supporting the theory that when horses are shown an open space they would like to reach but are unable to get to, they can potentially experience an increase in stress.

One stable design that increases contact opportunity between neighbouring horses is termed the ‘social-box’, which consists of two vertical bars, 2.5 m high and 0.3 m apart in a wall of a stable, allowing for interaction between adjacent horses. This design has been trialled with unfamiliar stallions driven in pairs to see if the increased opportunity of housed social interaction reduced unwanted interactions when working. Unwanted interactions during work were monitored prior to and post housing in adjacent social boxes (Gmel *et al*. 2022). Unwanted interactions during work reduced significantly when stallion pairs were housed in neighbouring social boxes, interpreted as a more compliant response to driver instruction during work, and continued to reduce throughout the treatment period, possibly due to habituation or adaptation.

Human interaction tests were used to assess the effect of bars being placed at the front of a stable, allowing stallions to eat haylage from the corridor and provide visual contact with conspecifics when foraging, a behaviour that would naturally occur in the wild (Søndergaard & Halekoh 2003). Horses in traditional housing were seen to approach the human quicker, be more approachable, and were less affected by being placed in an unfamiliar environment, however the bond between horses was stronger in the social setting. This implies that single housing may enhance the human-horse relationship and social housing has the benefit of improved social bonds between horses (Søndergaard & Halekoh 2003). As already mentioned, the impacts of such findings related to housing on the behaviour of horses in training or work, particularly when separated from herd mates when housed in a social setting, warrants further investigation.

### Natural housing of horses

Natural housing that aims to replicate the species-specific natural habitat, encouraging movement and social interaction whilst providing control and convenience for the owner, are well established across Europe and growing in popularity in the UK. The number of studies comparing these systems with other housing methods is limited, but those that exist tend to carry out data collection over an extended period. Marlinani *et al.* (2020) measured the day-time activity budgets of horses in what they termed an “*ethological housing system*” and demonstrated that they were similar to those reported for free-roaming feral horses. Placci *et al.* (2019) reported positive physiological indicators of welfare in naturally managed horses, with lower cortisol to dehydroepiandrosterone (DHEA) ratio, suggesting positive long-term effects of this housing style. However, horses housed overnight with day-time turn-out showed no difference in cortisol levels when compared to natural management, it being proposed that this could be due to the sleep quality being improved when horses were inside at night thus improving well-being (Mazzola *et al*. 2021). The most technologically advanced forms of natural housing, referred to as active systems, incorporate functional elements, such as individual, transponder-controlled automatic hay and concentrate feeders, drinking stations, access-controlled grazing and purpose-built rest areas, separated by distance or track systems to encourage movement. Hildebrandt *et al.* (2021) assessed the visit frequency for different functional elements, informing their placement in active systems when horse movement stimulation is a desired outcome of the design. Rose-Meierhöfer *et al*. (2010) reported that the highest movement level in a paddock was lower than the minimal value of an active design system, which was set up with functional elements distributed to encourage movement. Evidence therefore suggests that natural housing systems provide significant welfare benefits for horses and that the automated and access-controlled elements that are features of active systems could serve to address some of the negative owner perceptions and management challenges sometimes associated with the group housing of horses (Hartmann *et al.* 2012). The increased horse movement that can be stimulated as a result of active system design could also provide benefits in terms of exercise, exercise recovery, body condition management, and other health-related benefits. More studies into these systems are therefore needed.

### Housing assessed via health-related parameters

Endoscope results revealed greater accumulation of tracheal mucus in horses housed in enclosed stables (Millerick-May *et al*. 2012) and horses that were stabled experienced an increase in exhaled breath condensate pH and gaseous ammonia compared with those out in a paddock (Whittaker *et al*. 2010). Housing design can influence air quality; runner stables, which are designed for young horses or mares with foals, exceeded allowable bacteria levels by three times compared to individual stables (Grzyb *et al*. 2022), with results attributed to cleanliness level. Runner stables were cleaned once a month, in comparison with individual boxes that were cleaned once a week. It has been previously reported that total airborne particulate level was correlated with number of horses housed in the same barn (Rosenthal *et al.* 2006).

Junkkari *et al.* (2017) reported no difference in the occurrence of respiratory disease in weanlings housed in stables or loose-housing systems in cold conditions. They note that the incidence of respiratory conditions was higher in the youngest weanlings regardless of housing type and access to outdoor space. They also noted that Finn horses were better suited to cold climate housing than Standardbreds in terms of maintaining body condition, highlighting the need to consider the broader housing needs of horses in relation to welfare.

A “*low dust*” stable free of hay and straw achieved lower endotoxin levels compared to a conventional stable with hay and straw, but not lower than a paddock (McGorum *et al*. 2010). These results show that air quality cannot be attributed to housing design alone but also to overall management including feeding and bedding systems. The location of stables can also influence particulate matter levels. A slightly open stable, placed next to a racetrack road and car park, and an enclosed stable next to a manure handling building, racetrack road and city road, had a higher particulate matter than an enclosed stable next to a car park and woodland (Millerick-May *et al*. 2011). However, there are many factors that may affect these results, including foot traffic on the yard, forage type and provision method and activity of horses. These results, in part, support those of Berndt *et al.* (2010) who stated certain sources may produce endotoxins, such as manure, which could be another reason for higher levels in the enclosed stable next to manure storage.

Technological developments have led to improved mechanical ventilation, bringing air in from the outside into the centre of stables and distributing it through the ceiling into boxes, resulting in a decrease in CO_2_, ammonia and reduced respiratory mucus, but no difference in dust levels. Some results report seasonal variation, possibly because the ventilation is regulated by the indoor temperature of the stables, meaning it runs at a lower capacity during colder temperatures to prevent the stables falling below desired temperatures (Wålinder *et al*. 2011). Future research could investigate combining stall architecture with mechanical ventilation to maintain higher air quality, especially for winter periods when most windows are closed.

Immunological measures have been included in some housing studies with mixed findings. The relationship between CD4:CD8 lymphocytes was significantly higher in pregnant mares living in a reserve than in individual stabling, indicating higher immune cell activity. However, variations in breed or nutrition could have confounded these findings (Krakowski *et al*. 2017). In a more controlled study, it was reported that immune activation may be a result of higher endotoxins being present in the stable environment when compared with pasture (Berndt *et al*. 2010). However, reduced immunity could be due to an increase in stress. Relocation to single housing led to longer-lasting changes to immune cells than cortisol, suggesting stress having a stronger or more sustained effect on the immune system than the endocrine system (Schmucker *et al*. 2022). Marr *et al.* (2020) reported an increase in cortisol when stabling horses for 48 h. Horses also switched to a predominant left-limb preference in laterality suggesting an increase in sympathetic nervous system activation. However, IgA levels remained unchanged, potentially because the stress was not chronic enough to stimulate any immunological changes. Schmucker *et al.* (2022) had an extra step in their methodology which may have increased the stress, by splitting the group into two and then re-grouping before splitting into stables. The disruption of the stable grouping may have influenced stress and therefore the immune system in conjunction with the individual stabling.

Mach *et al.* (2021) reported changes in gut microbiota and positive behavioural responses for horses turned out to pasture for a period of one month and also identified microbiota species that appeared linked with behaviours indicating poor welfare in housed horses. Whilst the authors acknowledged that causality could not be inferred by the study, impacts of environment and/or diet change on gut microbiota composition and the effects of this on behaviour and welfare is an emerging and important area of science that warrants further investigation.

Molinari *et al*. (2020) explored the use of a number of oxidative stress markers as measures of positive or negative welfare in different housing environments and in relation to the presence or absence of stereotypies, but there was no significant difference in the parameters measured. Mal *et al*. (1991) attempted to identify a number of physiological markers between isolated, confined and pasture-housed mares to assist welfare evaluation. There was a variable age- and temperament-related response to a phytohaemagglutinin (PHA) skin test in the isolated mares, but no other housing or age groups. There was no difference in leukocyte response to the PHA test or adrenal response to a subsequent exogenous adrenocorticotrophin administration between any groups indicating that the housing types tested only elicited the mildest of physiological stress response, or no response at all.

### Animal welfare implications

The ways in which horses are housed can have a serious impact on their welfare. A significant and growing body of work now exists that aims to better understand this. This review consolidates the findings of this work and highlights gaps and opportunities to inform the future direction of this important area of horse welfare research, with a focus on encouraging work that has a more global perspective and serves to inform practical improvements that can be made to the ways in which horses are kept.

## Conclusion

The amount of research investigating the impacts of housing on horse welfare is growing, with 52% of reviewed papers published in the last eight years. Most studies identified in this review have been carried out in Europe and the USA, limiting global perspective. Whilst the housing of horses may not be as prevalent in some of the unrepresented nations, it is in others and the lack of coverage may well be limiting understanding and appreciation of country- or environment-specific implications. Behaviour analysis was the most frequently applied assessment and predominantly focuses on negative behaviours. However, as a species, horses do not reliably express negative behaviours in a coping situation (Budzyńska 2014) and so caution is needed in the interpretation of negative results as passive coping could provide an underestimation of effect. There is an increasing trend in the use of positive behaviours in housing assessment, good welfare is not just the absence of negative experience but also the display of positive welfare parameters. Including positive outcomes in future assessments of horse housing has the potential to add significantly to knowledge and is to be encouraged. Physiological measures of welfare began in the early 1990s and a combination of behavioural and physiological assessment in studies is now more common. Where physiological differences exist between housing types, they agree with behavioural findings, though physiological differences are not always identified when behavioural differences are found. This could be due to differences in housing type not being significant enough to elicit a physiological response, horses already having a high baseline of physiological response, studies not being carried out for a long enough duration to measure effects, or the chronic effects of the housing leading to coping responses that do not involve cortisol. Most studies in this review were relatively short in length, increasing study duration would give greater insight into the habituation process and longer-term effects of housing. Despite relatively simple definitions, measuring and diagnosing stress in humans and non-human mammals is challenging, necessitating the use of more than one measure and an appreciation of the nature of the species studied and the context within which the potential stressor is being measured for results to be correctly interpreted. In this vein, the diversity of experimental design between studies in terms of housing type, measures used, number and grouping of horses and duration of study add complexity to trend identification and a number of outputs would have been improved by providing more detail on experimental design and the specific nature and design of the housing being studied. Some studies investigated more fundamental impacts of housing on health, particularly respiratory health, immune function and changes to gut microbiota with variable but interesting findings. Some of these are directly linked with the design or siting of the housing, and some have links with physiological responses to long-term stress identified in other species. Generally, studies agree that keeping horses in groups outdoors is preferred and, where not possible, a proportion of time spent in an outdoor environment is recommended but must continue as part of a management routine to maintain the positive benefits for the horse. Space, social contact with conspecifics and improved ventilation are all important housing design or modification factors that positively impact behaviour and/or physiological health, ultimately improving horse welfare, and further studies to demonstrate successful incorporation into housing design are needed. A number of studies highlight the importance of considering factors beyond the housing design, such as feed, bedding, climate, horse age, breed and temperament, some of which have additional health implications and all of which can confound results. Such considerations, along with studies seeking to improve horse welfare in existing housing systems, in the face of limited space or within other management constraints, are of high value to the end user as a form of knowledge exchange that will impact welfare-friendly horse housing change.
